# Mixed lineage leukaemia histone methylases 1 collaborate with ERα to regulate HOXA10 expression in AML

**DOI:** 10.1042/BSR20140116

**Published:** 2014-12-08

**Authors:** Jie Yao, Li-Chao Fang, Zai-Lin Yang, Hui Huang, Yan Li, Jun Deng, Junsong Zheng

**Affiliations:** *Department of Laboratory Medicine, Southwest Hospital, Third Military Medical University, Chongqing 400038, People's Republic of China; †Department of Hematology, Southwest Hospital, Third Military Medical University, Chongqing 400038, People's Republic of China

**Keywords:** AML, oestrogen receptor, gene regulation, HOXA10, mixed lineage leukaemia, ALL, acute lymphoblastic leukaemia, AML, acute myeloid leukaemia, ChIP, chromatin immunoprecipitation, ER, oestrogen receptor, ERE, oestrogen response element, H3K4, H3 at lysine 4, HMT, histone methyltransferase, LSD1, lysine-specific demethylase 1, MSP, methylation-specific PCR, MLL, mixed lineage leukaemia histone methylase, NP40, Nonidet P40

## Abstract

HOXA10, a homeobox-containing gene involved in definitive haematopoiesis, which implicated in the pathogenesis of AML (acute myeloid leukaemia), has been studied extensively. But the regulatory mechanism that drives HOXA10 expression is still unclear. In the present paper, HOXA10 regulated by MLL1 (mixed lineage leukaemia histone methylase 1) with an epigenetic way has been demonstrated. The HOXA10 promoter contains several EREs (oestrogen response elements), including ERE1 and ERE2, which are close to the transcription start site, and are associated with E2-mediated activation of HOXA10. It has been shown that knockdown of the ERα (oestrogen receptor α) suppresses E2-mediated activation of HOXA10. Similarly, knockdown of MLL1 suppresses activation of HOXA10 and is bound to the ERE of HOXA10 promoter in an E2-dependent manner by forming complex with ERα. Knockdown of ERα affects the E2-dependent binding of MLL1 into HOXA10 EREs, suggesting critical roles of ERα in recruiting MLL on the HOXA10 promoter. More interestingly, the methylation status of histone protein H3K4 (H3 at lysine 4) with E2 is much higher than without E2 treatment in leukaemia cell. On the contrary, the methylation status of HOXA10 promoter with E2 treatment is much lower, which elevate the HOXA10 expression. Moreover, with ERα knockdown, the H3K4 methylation level is also decrease in myeloid cell. Overall, it has been clearly demonstrated that HOXA10 is transcriptionally regulated by MLL1, which, in coordination with ERα, plays a critical role in this process with epigenetic way and suggests a potential anti-E2 treatment of AML.

## Abstract 2

We find that E2 could elevate HOXA10 expression. We examine HOXA10 regulated by MLL1 with an epigenetic way. MLL1 bind to HOXA10 promoter through formatting complex with mainly ERα.

## INTRODUCTION

Homeobox-containing genes are key players in embryogenesis and development. Misregulation of homeobox genes is associated with tumourigenesis. More than 200 homeobox-containing genes have been identified in vertebrates, and they have been classified into two major groups, class I and II which encode transcription factors regulating pattern formation, differentiation and proliferation [1]. There is considerable evidence linking deregulated HOX expression with human acute leukaemia. This includes translocations targeting specific HOX genes, particularly those located at 5′ in their clusters (for example, HOXA9), or myeloid–lymphoid leukaemia, which normally functions to maintain proper HOX levels [2].

HOXA10 is a homeodomain transcription factor that is expressed in the most primitive haematopoietic cell compartment, and overexpression of HOXA10 leads to impaired lymphoid development *in vitro*. Moreover, overexpression of HOXA10 in murine bone marrow induces a myeloproliferative disorder characterized by expansion of the committed myeloid progenitor population (common granulocyte/monocyte progenitors or GMP) [3]. This myeloproliferative disorder evolving to AML (acute myeloid leukaemia) over time in HOXA10-overexpressing mice, demonstrates that HOXA10 acts as an important regulator of haematopoiesis, governing both proliferation and differentiation of hematopoietic progenitor and stem cells, where distinct fate outcomes depend on the HOXA10 concentration [4–6]. Although HOXA10 is a critical player in differentiation of myeloid cells and leukaemiagenesis, little is known about its own regulation.

HMTs (histone methyltransferases) are key enzymes that post-translationally methylate histones and play critical roles in gene expression, epigenetics and cancer. MLLs (mixed lineage leukaemia histone methylases) are human HMTs, such as MLL1, MLL2, MLL3 and MLL4 that specifically methylate histone H3K4 (H3 at lysine 4) and are linked with genes [7]. MLL gene is a frequent target for recurrent chromosomal translocations found in approximately 10% of human acute leukaemias, which can be defined as AMLs, ALLs (acute lymphoblastic leukaemias) [8]. Studies have demonstrated that several MLLs act as coregulators for E2-mediated activation of E2-sensitive genes. Some MLL-like methyltransferase complexes have been shown to directly interact with site-specific DNA-binding transcription factors [9,10].

Moreover, previous studies have showed there are ERE on promoter of HOXA10 gene [14], which means that ER possibly is regulator of HOXA10. We were interested in determining whether MLL act as coregulator for HOXA10 expression. In the present study, we report that MLL1 act as coregulator of ERα to regulate expression of HOXA10 in an epigenetic way.

## MATERIALS AND METHODS

### Myeloid cell line culture, E2 treatment, and antisense oligonucleotide experiments

HL-60, THP-1 leukaemia cell lines were maintained in RPMI 1640 containing 10% (v/v) FBS. The cells treated with a freshly prepared solution of the cells were grown up to 70% confluency and treated with different concentrations (0–1000 nM) of E2 at different time. The cells were then harvested and subjected to either RNA and protein extraction or ChIP (chromatin immunoprecipitation) assay.

For treatment of cells with antisense oligonucleotides, cells were grown up to 60% confluency in 60 mm culture flasks and transfected with varying amounts (3–9 μg) of different antisense oligonucleotides. Briefly, cocktails of different amounts of antisense oligonucleotide and transfection reagents (ifect, MoleculA) were made in the presence of 300 μl of the culture medium (without supplements) by incubating for 30 min, as instructed by the manufacturer. Cells were washed twice with the supplement-free culture medium, and finally submerged in 1.7 ml of medium (without supplements). The antisense oligonucleotide/transfection reagent cocktail was applied to the cells and incubated for 7 h before the addition of 2 ml of culture medium with all supplements and 20% activated charcoal-stripped FBS. The cells were then incubated for an additional 24 h before being treated with E2.

### RNA isolation and RT–PCR

Cells were washed and lysed with guanidine isothiocyanate solution. Total RNA was extracted using RNA Extraction Kit for Culture Cells (Takara) following the instruction manual. Primers for β-actin were chosen specifically to cross one intron in the β-actin gene. In the presence of contaminating genomic DNA, additional larger bands would be amplified; the lack of amplification of the larger band was used as a control to rule out contamination with any genomic DNA. The sequences and PCR cycling conditions were listed in Table 1.

**Table 1:** Primers used for RT–PCR, MSP, ChIP and antisense oligonucleotide experiments

Primer	(5′→3′)
RT-HOXA 10-F	CCCTACACGAAGCACCAGACACT
RT-HOXA 10-R	GCGTCGCCTGGAGATTCATC
β-Actin-F	AAGGCCAACCGCGAGAAGAT
β-Actin-R	TCGGTGAGGATCTTCATGAG
MLL1 antisense	TGCCAGTCGTTCCTCTCCACa
MLL2 antisense	ACTCTGCCACTTCCCGCTCAa
MLL3 antisense	CCATCTGTTCCTTCCACTCCCa
MLL4 antisense	CCTTCTCTTCTCCCTCCTTGTa
ERα antisense	CATGGTCATGGTCAGa
ERβ antisense	GAATGTCATAGCTGAa
Scramble antisense	CGTTTGTCCCTCCAGCATCTa
ERE1-F	GGATCAACGGACTAGGGGAGA
ERE1-R	CCTCAAAAGTGGCGAACCTG
ERE2-F	ACGGACTAGGGGAGAAAAGTG
ERE2-R	CCAGGAGACGCACCCCGACA
RT-MLL1-F	AACGGTTTCAGCTGCCTCTA
RT-MLL1-R	TTTGGGTCACCTGAACTTCC
HOXA10-Mr	ATAAGTTTATTAATCGGCGAAGTTC
HOXA10-Mr	AATAAAAAAAACAAAAAAAACCGAT
HOX10-Uf	AAGTTTATTAATTGGTGAAGTT
HOXA10-Ur	CCAATAAAAAAAACAAAAAAAACCA

### DNA extraction and MSP (methylation-specific PCR)

Genomic DNA was extracted from leukaemia cell lines and the whole blood of leukaemia patients and healthy volunteers with the QIAamp DNA Blood Mini Kit (Qiagen, Valencia, CA, U.S.A.) following the manufacturer's instructions. Sodium bisulfite modification of genomic DNA was performed with EZ DNA Methylation kit (Zymo Research) following the manufacturer's instructions, in which 2 μg of original genomic DNA was eluted in 20 μl of M-Elution buffer. The bisulfite-converted genomic DNA was amplified by MSP using the primer sets listed in Table 1 to analyse the promoter CpG methylation of HOXA10. MSP was performed in a volume of 25 μl containing 5 pmol of a primer set, 1.0 μl of bisulfite-converted DNA solution in M-Elution Buffer, and contents of *ExTaq* HS DNA Polymerase kit (TaKaRa) at the suggested concentrations (including 0.625 units of *ExTaq* HS DNA Polymerase per 25 μl of reaction mixture, 1×*ExTaq* Buffer [Mg^2+^ plus] and 250 μM of each dNTP).

### Nuclear protein isolation and Western blot

The whole-cell lysate was prepared in 1 ml of ice-cold buffer [50 mM Tris/HCl (pH 7.4), 150 mM NaCl, 5 mM EDTA, 0.5% (v/v) NP40 (Nonidet P40), 1 mM phenylmethylsulfonylfluoride, 10 μg/ml aprotinin, 10 μg/ml leupeptin]. The lysate was rotated 360° for 1 h at 4°C followed by centrifugation at 12 000× ***g*** for 10 min at 4°C to clear the cellular debris. Proteins were quantified using the BCA protein assay kit (Pierce Chemical Co.). Nuclear protein was extracted using Qproteome Nuclear Subfractionation Kit (Qiagen) by following the manufacturer's instructions. Nuclear protein was resolved on SDS/PAGE gels and transferred to nitrocellulose membranes, and Western blot analyses were performed using antibodies of ERα and ERβ (R&D Systems), anti-histone H3 (di-methyl K4) antibody(abcam), MLL1 antibody (Bethyl), anti-histone H3 antibody (Bethyl).

### ChIP assays

ChIP assays were performed using an EZ Chip chromatin immunoprecipitation kit (Upstate). In brief, cells were fixed in 4% (v/v) formaldehyde, lysed and sonicated to shear the chromatin. The fragmented chromatins were pre-cleaned with protein Gagarose and subjected to overnight immunoprecipitation with specific antibodies for ERa (oestrogen receptor a) and MLL1. Immunoprecipitated chromatins were washed and deproteinized, and DNA fragments were purified by phenol/chloroform extraction followed by precipitation overnight at −80°C. The purified DNA fragments were then used as templates in PCR amplification of four EREs of the HOXA10 promoter, using the primer pairs listed in Table 1.

### Coimmunoprecipitation of MLL–ER complexes

In order to confirm physical interaction of MLL and ERa, coimmunoprecipitation from HL-60 cells in the absence and presence of E2 was performed. In brief, cells were treated with 100 nM E2 for 6 h, and harvested for preparation of nuclear extract. E2-treated and untreated nuclear extracts were incubated overnight at 4°C with MLL1 antibodies bound to the protein agarose-G beads. The beads were separated, and washed with buffer C (20 mm Tris/HCl, pH 7.9, 5 mm MgCl_2_, 420 mm KCl, 0.5 mm dithiothreitol, 0.2 mm phenylmethanesulfonyl) in the presence of 0.1% NP40. The affinity-bound proteins were eluted from the beads using 0.2 m glycine (pH 2.9), and analysed by Western blot, using specific antibodies, for the presence of ERa.

### Cell apoptosis

Apoptosis was assessed using annexin-V conjugated with FITC. 1−5×10^6^ HL-60, THP-1 cell lines were treated with and without LSD1 (lysine-specific demethylase 1). And Cells were plated at 2.5×10^5^ per 60 mm dish in triplicate. After treatment, cells were washed twice with PBS, and stained with PI and FITC–annexin-V (Apoptosis & Necrosis Quantification Kit, Biotium Hayward) for 15 min in the dark. Cells were immediately analysed on GUAVA flow cytometer for PI and FITC–annexin-V staining.

### Data statistics

Results are expressed as the means±standard deviation. The *t* test and approximate calculation by normal distribution were used for studying the difference between groups. A *P* value less than 0.05 (double-sided) was considered as significant, and a *P* value less than 0.01 was considered as very significant.

## RESULTS

### HOXA10 expression elevate by E2

**Figure 1:**
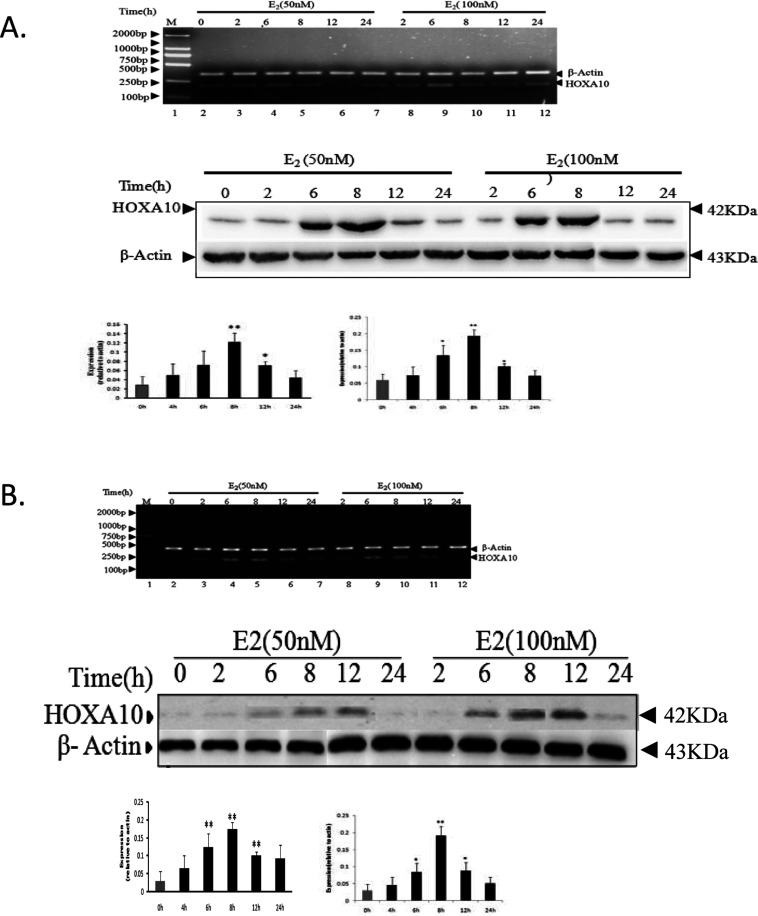
Effect of oestrogen on HOXA10 expression HL-60 (**A**) and THP-1 (**B**) cell were initially grown in the phenol red-free medium, and treated with different concentrations (50 and 100 nM) of E2 for (0–24 h), tamoxifen treatment +E2 at different time points as control. The total RNA and protein was isolated and analysed by RT–PCR and Western blot, using primers specific for HOXA10, β-actin was used as control. Quantification of Western-blot products is showed and The *t* test was used to examine the difference between groups.*P<0.05 and **P<0.01 compared between groups.

In order to determine whether HOXA10 is regulated by E2, a steroidogenic human cell line (HL-60 and THP-1 cells cultured in phenol-red free medium containing activated charcoal-treated FBS) was treated with different concentrations (50 and 1000 nM) of E2 for 8 h. The RNA was isolated from the E2-treated cells and analysed by RT–PCR, using HOXA10-specific primers (Table 1). Interestingly, the results demonstrated that HOXA10 was overexpressed upon exposure to E2 in a concentration-dependent manner (Figure 1). In comparison with the control, HOXA10 expression was significantly enhanced in all cell lines. It was found that 100 nM was the optimal concentration for the E2-mediated induction of HOXA10. Time-dependence experiments demonstrated that HOXA10 activation was maximized after 6–8 h of E2 treatment.

### ERα plays a critical role in E2-induced HOXA10 expression

**Figure 2:**
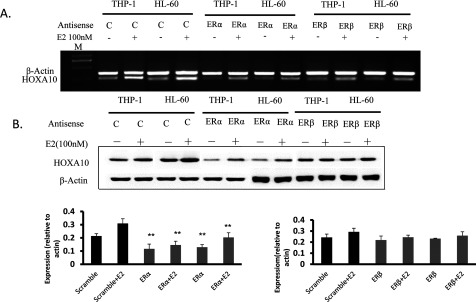
Effect of ERa and ERβ on E2-induced expression of HOXA10 HL-60 and THP-1 cells were grown up to 60% confluency and then separately transfected with phosphorothioate oligonucleotides specific for ERα and ERβ using Lipofectamine 2000 (Invitrogen). Control cells were treated with a scramble antisense oligonucleotide with no homology with the ERa and ERβ genes. The antisense oligonucleotide-transfected cells were incubated for 24 h and then treated with E2 (100 nM) for an additional 8 h. Cells were harvested and total RNA and protein was isolated and analysed by RT–PCR and Western-blot. The *t* test was used to examine the difference between groups.*P<0.05 and **P<0.01 compared between groups.

In order to study the potential role of ERs in E2-induced activation of HOXA10, ERa and ERβ were knocked down separately, using the specific antisense oligonucleotides, in HL-60 and THP-1 cells and exposed to 100 nM E2 for an additional 8 h. A scramble antisense oligonucleotide (with no homology to ERs) was used as a negative control. The results demonstrated that application of ERa antisense oligonucleotide knocked down the respective genes efficiently, at both the mRNA and the protein level. After confirming effective knockdown, the RNA from these ER knockdown and E2-treated cells for the expression levels of HOXA10 were analysed using RT–PCR. Interestingly, upon knockdown of either ERa, HOXA10 expression was suppressed (Figure 2). These results demonstrated that both ERa are essential for up-regulation of HOXA10.

### MLL1 plays critical roles in HOXA10 expression

**Figure 3:**
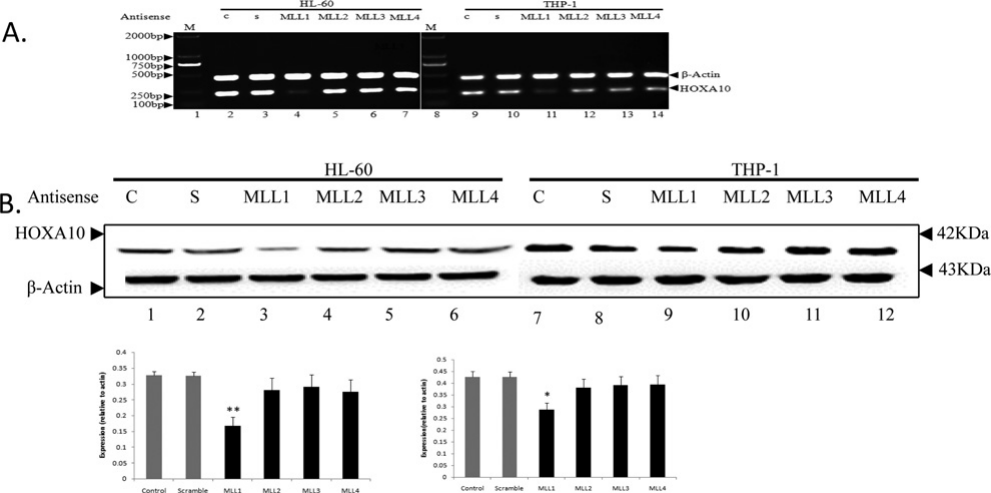
Effect of MLLs on expression of HOXA10 HL-60 and THP-1 cells were grown up to 60% confluency and then separately transfected with phosphorothioate oligonucleotides specific for MLL1, MLL2, MLL3 and MLL4 phosphorothioate oligonucleotides using Lipofectamine 2000 (Invitrogen). Control cells were treated with a scramble antisense oligonucleotide with no homology with the MLL1, MLL2, MLL3 or MLL4 gene. The antisense oligonucleotide-transfected cells were incubated for 24 h, and then cells were harvested and total RNA and protein was isolated and analysed by RT–PCR and Western blot. The *t* test was used to examine the difference between groups.*P<0.05 and **P<0.01 compared between groups.

As MLLs are well known as master regulators of HOX genes, and several MLLs are implicated in E2 signalling, whether any of the MLLs are involved in stimulation of HOXA10 expression was studied. MLL genes (MLL1, MLL2, MLL3 and MLL4) were knocked down separately using specific phosphorothioate antisense oligonucleotides. The efficiencies of different MLL (MLL1–MLL4)-specific antisense oligonucleotides and their most effective doses were studied. The specific MLL knockdowns were confirmed by analysing their respective gene expression at both the mRNA and protein levels. In parallel, a scramble antisense oligonucleotide without homology with any of the MLLs as a negative control was applied. As seen in Figure 3, upon application of MLLs antisense oligonucleotide, only the knocked down of MLL1 could induced that HOXA10 expression decreased significantly (Figure 3). Whereas, MLL2, MLL3 and MLL4 knockdown have no effect on HOXA10 expression. These results demonstrated that MLL1 played critical roles in the regulation of HOXA10.

### E2-induced recruitment of ERα and MLL1 in the HOXA10 promoter

**Figure 4:**
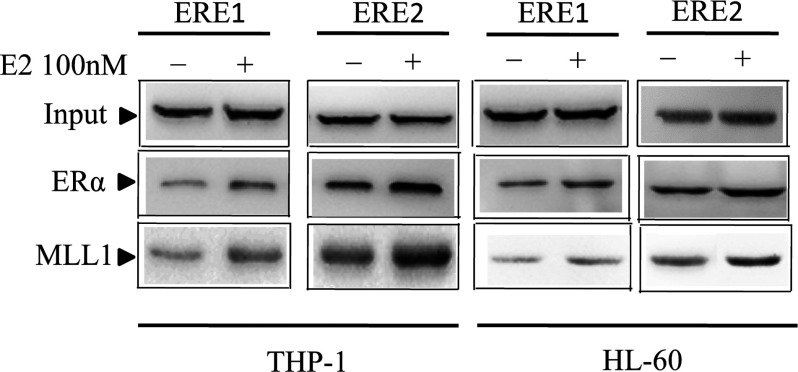
E2-dependent recruitment of ERa and MLL1 in ERE1 and ERE2 of the HOXA10 promoter HL-60 and THP-1 were treated with 100 nM E2 for 8 h. Afterwards, the control and E2-treated cells were subjected to ChIP analysis using antibodies against MLL1 and ERα. β-Actin antibody was used as control IgG. The immunoprecipitated DNA fragments were PCR amplified using primers specific for ERE1 and ERE2 of the HOXA10 promoter.

As the HOXA10 promoter contains several ERE1/2 regions within the first 3000 nucleotides upstream of the transcription start site, the involvement of some of these EREs (ERE1–ERE2) was studied by analysing the *in vitro* binding of ERs and MLLs. The *in vitro* binding of the different factors in the absence and presence of E2 were studied by ChIP assays, using antibodies against MLL1, ERα. ChIP experiments were also performed in parallel with the use of antibody against β-actin as a non-specific negative control. In brief, cells were treated with 100 nM E2 for 8 h. Afterwards the control and E2-treated cells were subjected to ChIP analysis. The immunoprecipitated DNA fragments were PCR amplified using primers specifically for ERE1 and ERE2 of the HOXA10 promoter. As seen in Figure 4, no significant binding of actin was observed in any of the EREs, irrespective of the absence and presence of E2. Binding of MLL1, ERα was increased in both ERE1 and ERE2 of the HOXA10 promoter with E2, compared with E2 treatment.

### Recruitment of MLLs to the HOXA10 EREs is mediated via ERα

**Figure 5:**
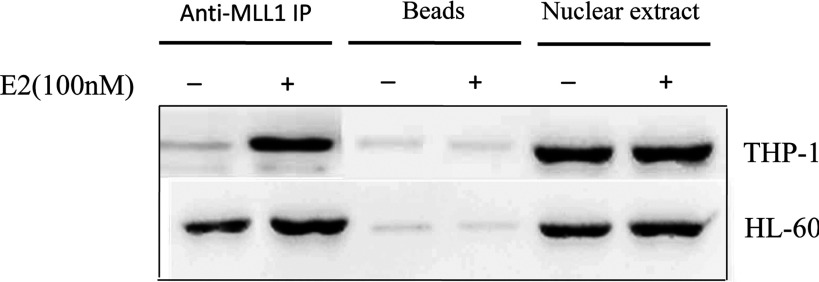
The interaction of MLL1 and ERs of HOXA10 promoter HL-60 and THP-1 cells were treated with 100 nM E2 for 8 h before being harvested for preparation of nuclear extract. The extracts were immunoprecipitated by using MLL1 antibody. The immunoprecipitated MLL1 complexes were then analysed by Western blot, using ERa antibodies. Immunoprecipitation with protein G agarose beads was used as negative control.

ERs are well known to bind directly to the EREs of the E2-responsive genes via their DNA-binding domains. MLLs (MLL1–MLL4) also have DNA-binding domains that might be involved in direct binding of promoters. This binding may be critical for regulation of basal transcription of the target genes. On the other hand, MLLs might be recruited to the HOXA10 promoter via protein–protein interactions directly or indirectly with ERs. Amino-acid sequence analysis demonstrated that MLL1–MLL4 had LXXLL domains [also called NR (nuclear receptor) boxes], which are known to be involved in E2-dependent interactions with ERs. The physical interactions of MLLs with ERα were further confirmed by using coimmunoprecipitation experiments. The interaction of MLL1 with ERa was studied separately. In brief, HL-60 cells were treated with 100 nM E2 for 8 h. Nuclear extracts were prepared from these E2-treated and untreated cells, and were incubated with MLL1 antibody (bound to protein-G agarose beads) overnight at 4°C. Proteins bound to the MLL1-attached and control beads were analysed by Western blotting using antibodies specific for ERa and MLL1. The results demonstrated that the interactions of both ERa with MLL1 were increased in the presence of E2 (Figure 5).

### H3K4 methylation status is increased by recruitment of MLL to the HOXA10 EREs

**Figure 6:**
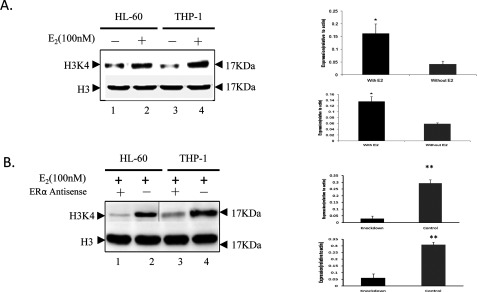
H3K4 methylation difference between with E2 and without E2 20 μg of protein were electrophoresed on SDS/PAGE (12% gel), transferred electrophoretically to nitrocellulose membranes, blocked with 5% (w/v) BSA, All results were confirmed by repeating on specimens from at least three separate preparations. Western-blot analysis was performed by using antibodies of H3K4. The *t* test was used to examine the difference between groups.*P<0.05 and **P<0.01 compared between groups.

As shown that MLL1 could bind to HOXA10 EREs to regulate HOXA10 expression through protein complex formation with ERα, the H3K4 methylation status with and without E2 treatment in HL-60 and THP-1 cells were studied since MLL contains specifically methylate histone H3K4. The H3K4 methylation significantly increased with E2 in both leukaemia cell lines (Figure 6A). On the contrary, the H3K4 methylation decreased with ERα knockdown (Figure 6B).

### The difference of H3K4 methylation status, HOXA10 promoter methylation status and HOXA10 expression with and without MLL1 knockdown

**Figure 7:**
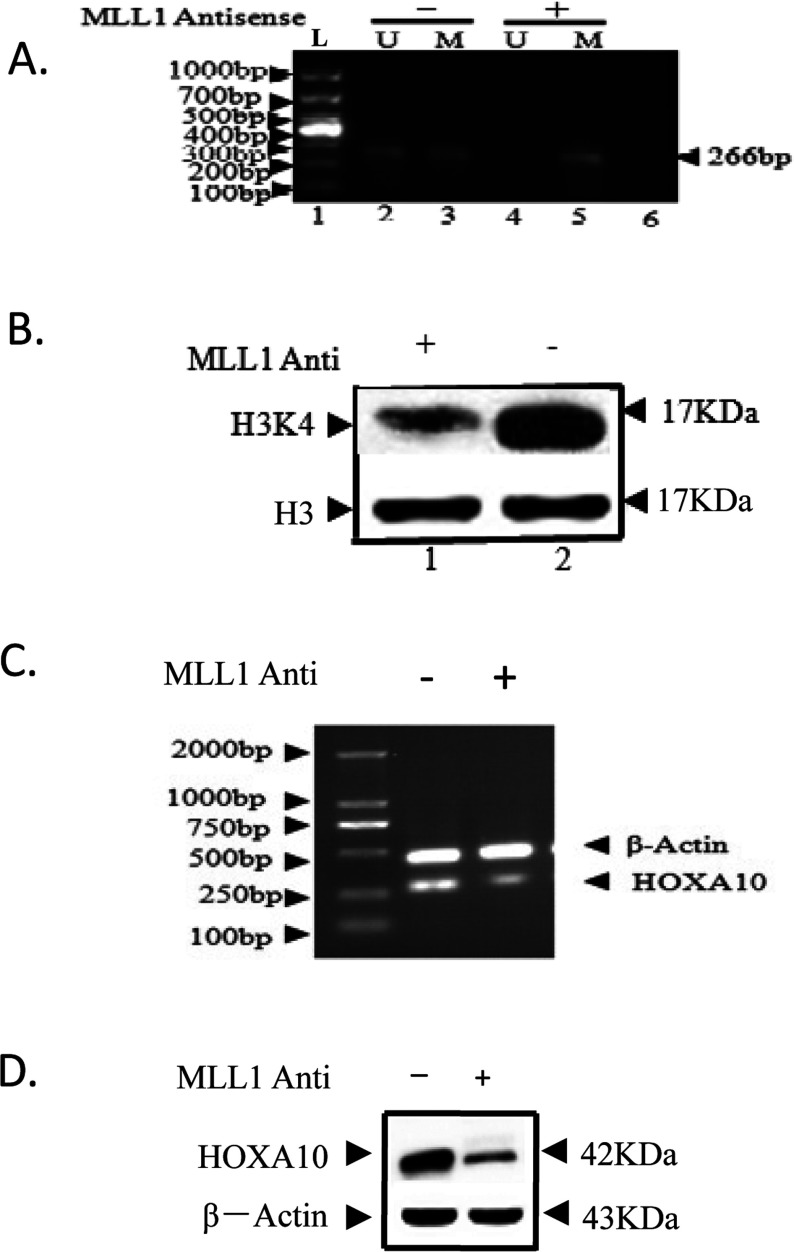
The difference of H3K4 methylation status, HOXA10 promoter methylation status and HOXA10 expression with and without MLL1 knockdown HL-60 was grown up to 60% confluency and then separately transfected with phosphorothioate oligonucleotides specific for MLL1 phosphorothioate oligonucleotides using Lipofectamine 2000 (Invitrogen). Control cells were treated with a scramble antisense oligonucleotide with no homology with the MLL1, MLL2, MLL3 or MLL4 gene. The antisense oligonucleotide-transfected cells were incubated for 24 h and then cells were harvested and DNA, total RNA and protein was isolated and analysed by MSP, RT–PCR and Western-blot to test HOXA10 promoter methylation status with and without MLL1 knockdown (**A**), Lane L was 1000-bp DNA ladder. Lane M and U referred to methylation and unmethylation bands of MSP, respectively. The gel images showed the promoter CpG methylation of HOXA10 promoter in acute leukaemia cell line. (**B**) H3K4 methylation status with and without MLL1 knockdown. HOXA10 expression level with and without MLL1 knockdown in mRNA (**C**) and protein (**D**).

MSP, RT–PCR and Western blot were performed to respectively analyse the methylation status of HOXA10 promoter CpG island, mRNA expression of MLL1 and HOXA10, protein expression and H3K4 methylation status in the leukaemia cell line (HL-60) with and without MLL1 knockdown (Figure 7).

With MLL1 knockdown, promoters of HOXA10 was fully methylated in HL-60, compared with semi-methylated without MLL1 knockdown (Figure 7C). However, the methylation status of H3K4 decreased with MLL1 knockdown (Figure 7D).

### Cell apoptosis

**Figure 8:**
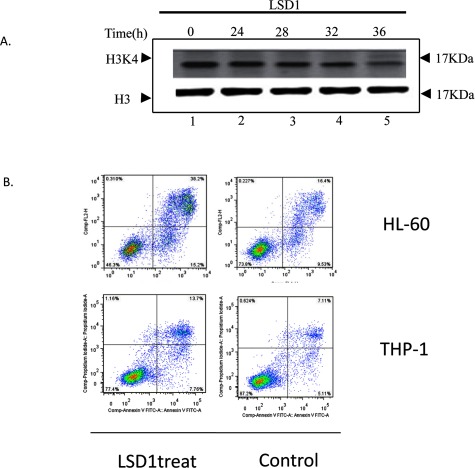
H3K4 demethylation induce apoptosis of AML cell (**A**) LSD1 2 μg in 20 μl cell reaction to treat cells for (24–36) h. (**B**) cells were treated by LSD for 36 h, then apply to flow cytometry.

Pi (propidium iodide) simple staining was performed to analyse the cell apoptosis on the HL-60 and THP-1 lines with or without LSD1 treatment. With the H3K4 demethylation treatment of LSD1, the cell apoptosis had significant increase in HL-60 and THP-1 cells (AML), compared with leukaemia cell line without LSD1 demethylation treatment (Figure 8).

## DISCUSSION

HOX genes play a major role in embryonic development. HOX gene products act as transcription factors that regulate critical genes that are necessary for cell differentiation and development. Hox proteins are transcription factors, which are proteins that are capable of binding to specific nucleotide sequences on the DNA called enhancers where they either activate or repress genes. The same Hox protein can act as a repressor at one gene and an activator at another gene [5,11].

Studies have been shown that HOXA10 is a homeodomain transcription factor that is frequently overexpressed in human AML. In murine bone marrow transplantation studies, HOXA10 overexpression induces a myeloproliferative disorder with accumulation of mature phagocytes in the peripheral blood and tissues. Over time, differentiation block develops in these animals, resulting in AML [12]. In general, ERs, along with various coregulators, are recruited to EREs present in the promoters of E2-responsive genes [13]. Studies have demonstrated that the HOXA10 promoter contains two EREs within 3000 bp upstream of the transcription start site that ERs could bind, which means ERs may play an important role in HOXA10 expression [14]. Indeed, the ER gene has been shown to be a growth and/or metastasis suppressor in multiple cell types, and the negative effect of oestrogen on haematopoiesis suggests that this is also the case in leukaemias [15]. And studies have suggested a potential role of ER in leukomogenesis because ER CpG island methylation at very high frequency caused inactivation of ER expression in human leukaemias. Also it has been shown that the inactivation of ER in mice leads to a leukaemia-like syndrome [16–18].

Oestrogens are important signalling molecules that regulate various physiological processes such as cell growth, reproduction, development and differentiation. They also have a role in several pathological processes of hormone-dependent diseases, including cancers. Cellular signalling by oestrogens is mediated via the ERa and ERβ (oestrogen receptor β), and the two ER's exert different biological responses. ERa serve as an oncogene, however, ERβ serve as tumour suppress gene. The metabolism of endogenous oestrogens can be associated with the development of hormone-dependent human cancers. Moreover, it has recently been demonstrated that some haematological malignancies, including leukaemia and lymphoma cells, are positive for oestrogen receptors (ERs). Furthermore, some lymphomas, including various subtypes of NHL (non-Hodgkin's lymphoma), develop with different frequencies between men and women, and suggested that E2 significantly increased the proliferation of Hut-78 T cell and Jurkat cell [19].

In the present study, we treated leukaemia cell lines with 100 nM E2, that concentration has been used by many studies [20,21] and proved by experiment. And there was a study found that the leukaemia cell line RMPI-8226 contained the highest levels of oestrogen (182.06 pg/106 cells) and 17b-estradiol (753.45 pg/106 cells), compared with breast and ovarian cancer. The present study shows the HOXA10 expression could be activated with E2 treatment for 8 h and antisense oligonucleotide-mediated knockdown of either ERa down-regulates the E2-mediated activation of HOXA10, indicating their critical roles in the process. ER-mediated regulation of E2-sensitive genes is a complicated process. In the presence of E2, ERs are activated and bind to the EREs of E2-responsive genes, eventually resulting in transcription activation. In addition to ERs, E2-mediated gene activation requires various other coregulators and coactivators that result in chromatin modification and remodelling. It demonstrates that MLL1 plays a crucial role in the regulation of HOXA10. Knockdown of MLL1 suppresses the activation of HOXA10.

There is sequence analysis demonstrated that the HOXA10 promoter contains two EREs that close to transcription start site. *In vitro* binding analysis (ChIP), it is demonstrated that, in the presence of E2, more MLL1 could bind to both ERE1 and ERE2, compared with absence of E2. Furthermore, the coimmunoprecipitation experiments demonstrate that MLL1 interacts with ERa in an E2-dependent manner. These results suggest that MLL1 could bind to HOXA10 promoter through conforming a protein complex with ERa.

To further understand the MLL1 mediated regulation of HOXA10 gene in AML, we study the methylation status of H3K4 with and without E2, and the methylation status of H3K4 and the methylation status of HOXA10 promoter with and without MLL1 knockdown. The level of H3K4 methylation with E2 is higher than that without E2. And the HOXA10 expression is also increased with E2 treatment. With MLL1 knock down, the level of H3K4 methylation significantly decreases and the methylation status of HOXA10 is fully methylated, which induces the HOXA10 expression to elevate significantly both in mRNA and protein.

Moreover, HL-60 and THP-1 cell with LSD1 demethylation treatment, the apoptosis is significantly increased. These above results suggest MLL1 is the key factor in regulation of HOXA10 and MLL1 regulates the HOXA10 expression in epigenetic way. MLL1 binds to promoter of HOXA10 through ERs to regulate HOXA10 expression, because MLLs possess H3K4-specific HMT activity to methylate H3K4, which could elevate HOXA10 expression.
