# Expression of Concern: Mixed lineage leukaemia histone methylases 1 collaborate with ERα to regulate HOXA10 expression in AML

**DOI:** 10.1042/BSR-20140116_EOC

**Published:** 2021-04-07

**Keywords:** AML, gene regulation, HOXA10, mixed lineage leukaemia, oestrogen receptor

The Editorial Office has been made aware of potential issues surrounding the scientific validity of this paper, hence has issued an expression of concern to notify readers whilst the Editorial Office investigates.

